# Laparoscopic treatment of a giant seminal vesicle cyst with hemorrhage: A case report

**DOI:** 10.1097/MD.0000000000026142

**Published:** 2021-05-28

**Authors:** Yuzhu Hou, Xuejiao Hu, Yixing Duan, Wubin Tan, Xi Guo

**Affiliations:** Hunan Provincial People's Hospital, The First Affiliated Hospital of Hunan Normal University, Changsha, PR China.

**Keywords:** seminal vesicle, Hemorrhage, laparoscopic

## Abstract

**Rationale:**

A seminal vesicle cyst is a benign lesion of the seminal vesicle that is usually asymptomatic. However, when a giant seminal vesicle cyst ruptures and bleeds, it can cause obvious clinical symptoms. To our knowledge, no single giant seminal vesicle cyst with hemorrhage has been reported in current studies, and surgery is the primary method to treat seminal vesicle hemorrhage with obvious symptoms.

**Patient concerns:**

A 31-year-old man presented with urination pain but without obvious urination frequency and urgency, dysuria, and discomfort. Rectal palpation in the chest-knee position revealed a hard mass palpable in the upper right with a smooth surface and mild tenderness, and the upper edge of the mass could not be palpated.

**Diagnosis:**

The results of the B-mode ultrasound indicated a mixed echogenic lump between the bladder and prostate, with a size of 81 × 76 mm. The computer tomography scan showed an “S” tubular lump in the right side of the pelvic cavity. The mass has a computer tomography value of 58 to 70 HU, and uneven reinforcement can be observed. On the basis of the results of the magnetic resonance imaging of the urinary bladder, the lump has T1 and T2 signals of equal lengths.

**Interventions:**

The patient was diagnosed with a huge right seminal vesicle cyst with hemorrhage and was treated via laparoscopic surgery.

**Outcomes:**

The patient recovered quickly after the operation, and the symptoms of urination pain were significantly improved.

**Lessons:**

Seminal vesicle hemorrhage is clinically rare, and laparoscopic treatment is an effective and safe surgical method for the treatment of seminal vesicle cysts.

## 1 Introduction

Spermatocysts are benign lesions of the seminal vesicles.^[1]^ The majority of patients with spermatocysts have no obvious clinical symptoms. The common symptoms include bloody seminal vesicles, hematuria, ejaculatory disturbances, and frequency and pain of urination. Patients with large seminal vesicles or acute bleeding may also present with abdominal pain, difficulty urinating, and even hemorrhagic shock.^[2]^ B-mode ultrasound is often used as the test of choice, but magnetic resonance imaging (MRI) has advantages over B-mode ultrasound and computer tomography (CT) scan in diagnosing seminal cysts and has a better ability to identify soft tissue and thus determine whether a cyst is bleeding.^[3]^ For small benign cysts, asymptomatic or mildly symptomatic, without pediatrics, conservative treatment is often used. Malignant cysts with significant clinical symptoms and a cyst with a diameter of ≥2.5 cm are considered for surgical treatment.^[4]^ Laparoscopic vesiculectomy is often considered the most effective and safest method. In this study, we report a case of a seminal cyst with hemorrhage treated laparoscopically for the first time. After surgery, the patient had relief from painful urination.

## 2 Case report

**Figure 1:**
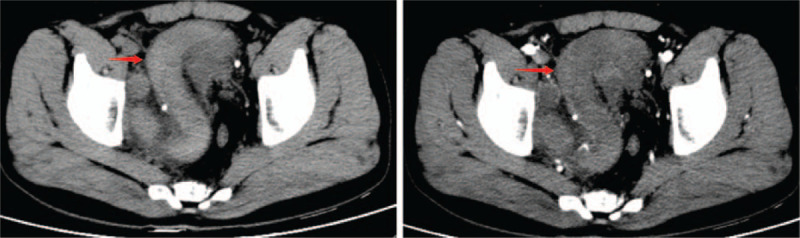
CT scan of the pelvis showing an “S” tube on the right side of the pelvis with a CT value of about 58 to 70 HU. The enhanced scan seemed to show a slight uneven enhancement, with slightly blurred fat spaces and a little exudation, and the prostate and seminal vesicle glands moved left under pressure.

**Figure 2:**
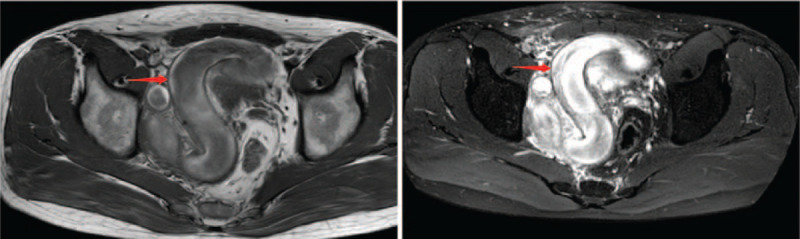
MRI scan of the pelvis showing a tortuous tube-shaped lesion with T1 and T2 signals of equal lengths on the right side of the pelvic cavity. A large amount of exudation can be observed around the lesion, and the boundary with the seminal vesicle gland on the right side is not clear. The enhanced scan shows the enhancement of the tube wall and its head end obturated to the right. The intermuscular space extends, and the prostate, rectum, and seminal vesicle glands are compressed and moved to the left.

A 31-year-old male was admitted because of “pain in urination for 7 days and pelvic mass found in B-mode ultrasound for 1 day.” Seven days ago, the patient had no obvious incentive to urinate, no hematuria, no urination frequency and urgency, and no waist pain. He had no obvious relief with self-administered antibiotics. One day before, B-mode ultrasound showed a mixed echogenic mass between the bladder and prostate. The patient had normal defecation and urine, no blood sperm, no special personal history, and no family genetic history. He married at the right age, and he has a son and a daughter. Specialist examination: no localized uplift, no mass, no percussive pain, and no vascular murmurs were found in both kidneys. No tenderness in the ureter stroke region, no localized eminence in the suprapubic region of the bladder, and no filling or tenderness in the bladder region was observed. External genitalia were normal, and normal testis and epididymis can be touched in both sides of the scrotum. Digital rectal examination of the rectum: in the chest-knee position, a hard mass could be touched in the upper right, with smooth surface, poor mobility, and mild tenderness. The mass was about 3 cm wide. The upper edge of the mass could not be touched. No blood stain was found in the finger sheath. Admission blood test routine: WBC count was 10.52 (×10^9^/L), and neutrophil percentage was 74.8 (%). Urine routine: urine color, yellow; leukocyte count, 14.1 (1/UL); epithelial cells, 17.4 (1/UL); urine protein, 1+; and nitrite and leukocyte esterase, negative. No obvious abnormalities were observed in liver and kidney function and prostate-specific antigen. CT scan: on the right side of the pelvic cavity, the “S” tubular structure was found, the CT value was about 58 to 70 HU, the boundary between the lesion and the bowel and bladder was clear, and the boundary between the lesion and the right genital accessory structure was not clear. The enhanced scan showed a slight uneven enhancement, and the surrounding fat space was slightly fuzzy. A little exudation was observed around, and the prostate and seminal vesicle glands were compressed to the left (Fig. 1). MRI scan: on the right side of the pelvic cavity, the “S” tubular structure was seen as a striated tubular signal focus of T1 and T2 of equal lengths. Considering that the right side of the reproductive accessory structure has a high possibility of origin, its internal bleeding is complicated by peripheral infection (Fig. 2).

Infection was controlled with reasonable antibiotics. The operation was performed under general anesthesia. Before laparoscopic surgery, transurethral seminal vesicle microscopy was conducted to further determine the cause of right seminal vesicle cyst formation. During the operation, verumontanum was found using Fr4.5 seminal vesicle microscopy, and the bilateral ejaculatory tubes were then examined. The left verumontanum could be observed with unobstructed left ejaculatory tube, whereas the right ejaculatory tube was blocked, so the right verumontanum could not be observed. It was confirmed that the seminal vesicle cyst was caused by right ejaculatory tube atresia.

**Figure 3:**
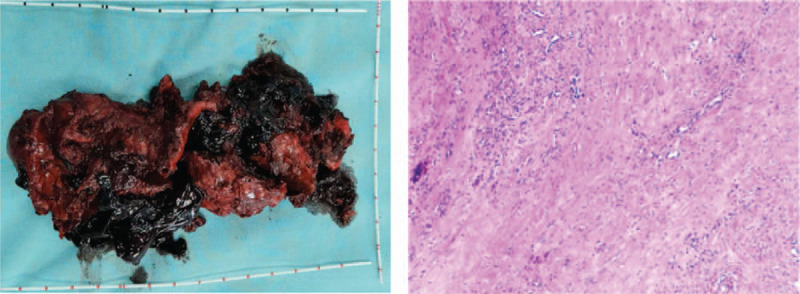
Image of a surgically removed cyst, showing a dark red blood clot.

Considering the large number of seminal vesicle cysts that were associated with hemorrhage, laparoscopic surgery was selected for the treatment of the cyst, which is currently the safest and most effective surgical method for the treatment of seminal vesicle cysts. With the right ureter as the reference, the retroperitoneum was opened, and the cysts were searched in the pelvis along the right vas deferens. The right sidewall of the bladder and the anterior space of the bladder were cut open with an ultrasound knife. A hard and fixed mass was observed at the rear of the right bladder. The ultrasound knife was used to cut open the cyst, and a dark red blood clot was found inside the cyst. The blood clot was about 500 mL, the cyst wall was removed in pieces using the ultrasound knife, and the necrotic tissue inside the cyst was cleared. The wound was rinsed with normal saline, and the specimen was taken out, with a size of about 12 × 7 cm (Fig. 3). Postoperative pathological results: (pelvic tumor) the cyst wall is composed of fibrous tissue without epithelium, and smooth muscle bundles were found in the local cyst wall. Combined with clinical and imaging findings, considering seminal vesicle cysts with hemorrhage and inflammatory cell infiltration. Immunohistochemistry: CK(pan) (−), Ki67 (+, 2%), CD34 (blood vessels +), SMA (smooth muscle +), desmin (smooth muscle +), β-catenin (scattered cytoplasm +), H-CALD (smooth muscle +), EMA (−), ERG (blood vessels +), and CD31 (blood vessels +).

**Figure 4:**
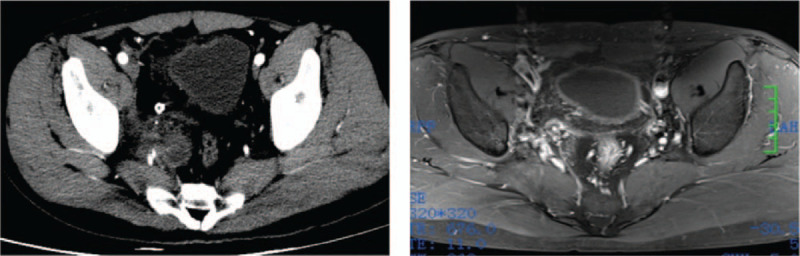
CT scan of the pelvis showing changes after cystectomy; a little cystic duct low-density shadow remains. MRI scan of the pelvis showing no recurrence of cysts after 3 months of surgical treatment.

The patient recovered well after surgery, and the symptoms of urination pain were significantly improved. The patient was discharged from the hospital on the 10th day after surgery. In the CT review of the pelvis showing changes after cystectomy, a little cystic duct low-density shadow remains. After 3 months of follow-up, no recurrence of the cyst, and no related postoperative complications were observed (Fig. 4).

## 3 Discussion

A seminal vesicle cyst is a benign lesion of the seminal vesicle, first reported by Smith in 1872, with an incidence rate of about 0.005%.^[1]^ It can be congenital and acquired naturally, which includes a seminal vesicle cyst and middle renal tubular malformation. The latter is often associated with other developmental malformations, such as ipsilateral renal insufficiency (Zinner syndrome),^[2]^ heterotopic vas deferens, pelvic heterotopic kidney, cryptorchidism, and hermaphroditism.^[3]^ It has also been reported to be associated with autosomal dominant polycystic kidney disease. About 60% of the cases of polycystic kidney disease are associated with seminal vesicle cysts.^[4]^ It can be acquired naturally because of ejaculatory tube atresia due to various causes after birth, such as inflammation, stones, and urethral and prostate surgery.^[5]^ In this case, the results of the CT and MRI examination are as follows: no deformity of the urinary system and reproductive system, in good health previously, no history of urethral surgery, and no family history of genetic disease. During the operation, seminal vesicle microscopic examination showed that the patient had right ejaculatory tube atresia, which was considered highly likely caused by inflammation. Most patients with this disease have no obvious clinical symptoms. Common symptoms include hemospermia, hematuria, ejaculation disorder, and urination frequency and pain. Patients with huge seminal vesicle cysts or acute bleeding may have abdominal pain, dysuria, etc. The most common symptoms of patients that visited a hospital are hemospermia or urination pain.

The diagnosis of seminal vesicle cyst should be combined with the examination of clinical symptoms, digital rectal examination, and imaging examination. Digital rectal examination is an important diagnostic method, which not only is comprehensive but also can understand the prostate and rectal conditions. B-mode ultrasound can be used as the first choice of examination. CT and MRI can not only show the relationship between the seminal vesicle cyst and the prostate and rectum more clearly but also classify the seminal vesicle mass as solid or cystic mass.^[6]^ CT findings of typical seminal vesicle cysts: local parenchymal cysts with cyst wall enhancement, but no intrathecal enhancement, and water sample density. And nonuniform enhancement in case of intracapsular hemorrhage. MRI showed low intensity on T1-weighted images and high intensity on T2-weighted images as well as increased intensity on T1-weighted images in case of intracranial hemorrhage or infection.^[7,8]^ In this study, owing to intracranial hemorrhage, the density of CT scan was higher than that of water, and uneven enhancement could be observed inside the cyst. MRI showed T1 and T2 signals of equal lengths. Compared with B-ultrasound and CT scan, MRI has more advantages in the diagnosis of the seminal vesicle cyst, and MRI has better recognition ability for soft tissue to determine the presence of hemorrhage inside the cyst.^[9]^

The treatment of seminal vesicle cysts was unified. Conservative treatment was adopted for patients with benign small cysts and with asymptomatic or mild symptoms and those who are childless. Surgical treatment was adopted for patients with malignant cysts, obvious clinical symptoms, and cysts with a diameter of >2.5 cm. Surgical methods can be divided into 3 categories^[10]^: traditional open surgery (suprapubic approach and perineal approach), transurethral seminal vesicle cyst resection, and laparoscopic seminal vesicle cystectomy. Seminal vesicle is located in the deep pelvic cavity, traditional open surgery field exposure is not well, and it has big trauma, slow recovery and postoperative complications. The laparoscopic approach is the safest and effective way to treat seminal vesicle cysts.^[11]^ To our knowledge, this is the first reported in current English literature that the laparoscopic treatment of simple giant seminal vesicle cysts with hemorrhage.

Carmignani first reported the laparoscopic removal of seminal vesicle cysts in 1995.^[12]^ The laparoscopic treatment of seminal vesicle cysts has the advantages of good surgical field exposure, small trauma, quick recovery, and less postoperative complications.^[13]^ In recent years, urology departments have performed an in-depth laparoscopic radical resection of prostate cancer and bladder cancer, and a surgeon can accurately identify the local anatomical structure and reduce the intraoperative damage to the rectum, bladder, vas deferens, and ureters. Laparoscopic resection is considered the safest and effective treatment method for giant seminal vesicle cysts. In this study, the patient was married, and he has 2 children. He had right ejaculatory tube atresia and a large and irregular cyst, so laparoscopic surgery was selected for the complete excision of the right seminal vesicle. With the gradual maturity of urethral endoscopy, more reports on the treatment of seminal vesicle cysts by urethral endoscopy have been conducted.^[14]^ In urethral endoscopy, the trauma is less than that in laparoscopy, and the patients recover more quickly. However, long-term follow-up is needed to further verify its postoperative efficacy and complications. Regular and healthy sex life, avoiding excessive drinking and smoking, and actively controlling urinary tract infections may play a role in preventing acquired seminal vesicle cysts. Early detection and early diagnosis are the key to preventing and treating the disease.

## Acknowledgments

We would like to thank the First Affiliated Hospital of Hunan Normal University (Hunan Provincial People's Hospital) for promoting the international cooperation.

## Author contributions

**Conceptualization:** Yuzhu Hou.

**Data curation:** Xuejiao Hu.

**Formal analysis:** Yuzhu Hou.

**Investigation:** Xuejiao Hu.

**Methodology:** Xi Guo.

**Resources:** Yixing Duan.

**Software:** Wubin Tan.

**Supervision:** Xi Guo, Yixing Duan.

**Validation:** Yixing Duan.

**Visualization:** Yuzhu Hou.

**Writing – original draft:** Yuzhu Hou.

**Writing – review & editing:** Yixing Duan.
